# Analysis of the quantitative balance between insulin-like growth factor (IGF)-1 ligand, receptor, and binding protein levels to predict cell sensitivity and therapeutic efficacy

**DOI:** 10.1186/s12918-014-0098-y

**Published:** 2014-08-13

**Authors:** Dan Tian, Pamela K Kreeger

**Affiliations:** 1 Department of Biomedical Engineering, University of Wisconsin-Madison, 1550 Engineering Dr, Madison 53706, WI, USA; 2 University of Wisconsin Carbone Cancer Center, 600 Highland Ave, Madison 53792, WI, USA

**Keywords:** Insulin-like growth factor (IGF), Mathematical modeling, Ovarian cancer

## Abstract

**Background:**

The insulin-like growth factor (IGF) system impacts cell proliferation and is highly activated in ovarian cancer. While an attractive therapeutic target, the IGF system is complex with two receptors (IGF1R, IGF2R), two ligands (IGF1, IGF2), and at least six high affinity IGF-binding proteins (IGFBPs) that regulate the bioavailability of IGF ligands. We hypothesized that a quantitative balance between these different network components regulated cell response.

**Results:**

OVCAR5, an immortalized ovarian cancer cell line, were found to be sensitive to IGF1, with the dose of IGF1 (*i.e.,* the total mass of IGF1 available) a more reliable predictor of cell response than ligand concentration. The applied dose of IGF1 was depleted by both cell-secreted IGFBPs and endocytic trafficking, with IGFBPs sequestering up to 90% of the available ligand. To explore how different variables (*i.e.*, IGF1, IGFBPs, and IGF1R levels) impacted cell response, a mass-action steady-state model was developed. Examination of the model revealed that the level of IGF1-IGF1R complexes per cell was directly proportional to the extent of proliferation induced by IGF1. Model analysis suggested, and experimental results confirmed, that IGFBPs present during IGF1 treatment significantly decreased IGF1-mediated proliferation. We utilized this model to assess the efficacy of IGF1 and IGF1R antibodies against different network compositions and determined that IGF1R antibodies were more globally effective due to the receptor-limited state of the network.

**Conclusions:**

Changes that affect IGF1R occupancy have predictable effects on IGF1-induced proliferation and our model captured these effects. Analysis of this model suggests that IGF1R antibodies will be more effective than IGF1 antibodies, although the difference was minimal in conditions with low levels of IGF1 and IGFBPs. Examining how different components of the IGF system influence cell response will be critical to improve our understanding of the IGF signaling network in ovarian cancer.

## Background

The insulin-like growth factor (IGF) network plays critical roles in development, normal tissue maintenance, and diseases such as cancer by regulating cell proliferation and survival [1]–[5]. The importance of the IGF network in development is clear as knockout mice for IGF ligands and receptors are embryonic lethal [6],[7], exhibit fetal growth restriction [8]–[11], or have shortened lifespans [12],[13]. Additionally, the IGF network is nearly ubiquitously expressed in solid and hematologic malignancies [14],[15]. Given the important role that IGF signaling plays in regulating cell behavior, it has emerged as a potential therapeutic target; however, due to its complexity, it remains unclear what is the optimal way to control this network.

The IGF network is composed of two ligands, IGF1 and IGF2, that are bound by two transmembrane receptors, type 1 IGF receptor (IGF1R) and type 2 IGF receptor (IGF2R) [16],[17]. IGF1R is a tyrosine kinase receptor that can bind both IGF1 and IGF2 to initiate activation of two principle downstream signaling pathways, PI3K/AKT and MAPK/ERK, leading to changes in cell proliferation, differentiation, and apoptosis [18],[19]. IGF-IGF1R complexes are internalized by receptor-mediated endocytosis and degraded by the lysosome or recycled back to the cell surface [20]–[22]. In contrast, IGF2R lacks an intracellular tyrosine kinase domain and only binds IGF2; as a result, it acts as a sink to regulate extracellular concentrations of IGF2 [23]. In addition to these interactions, the majority of IGF ligand circulating in the serum is bound to a family of six binding proteins (IGFBPs) [24],[25]. These ligand-binding protein interactions are of higher affinity than ligand-receptor interactions, preventing ligand-receptor binding unless disrupted by IGFBP proteases [26],[27]. While all IGFBPs bind to IGF ligands, prior studies have also seen that through this interaction, IGFBPs can actually potentiate IGF actions. For example, IGFBP5 overexpression in breast cancer cell models was found to have anti-proliferative and pro-apoptotic effects consistent with ligand sequestration [28], but the opposite was observed in other cancer models such as prostate cancer and retinoblastoma [29],[30]. Additionally, post-translational modifications such as phosphorylation can impact affinity of IGFBPs for IGF ligands, altering the effect of these proteins on cell behavior [31]. Finally, the IGF system has been found to crosstalk with the closely related insulin receptor (IR), and signaling-competent heterodimers of IGF1R/IR that behave analogously to IGF1R can form in cells expressing both receptors [32]–[35]. While it is recognized that these different processes (*i.e.*, trafficking, IGFBP sequestration, differential receptor-ligand interactions) can affect cellular behavior, they have not been subjected to systematic study to determine how they impact interpretation and application of experimental findings.

Understanding the impacts of these different processes may have clinical relevance, as epidemiological evidence suggests that the relative balance between IGF network components plays an essential role in maintaining healthy tissues. Indeed, alterations in network composition have been observed in multiple cancers, including ovarian cancer. For instance, patients with high circulating levels of IGF1 have an increased risk of developing ovarian cancer before the age of 55 [36],[37], and high levels of IGF1 mRNA and protein are further linked to disease progression [38]. Excess IGF1 has been shown to impact the ovarian surface epithelium of mouse ovaries, leading to hyperplasia and altered extracellular matrix deposition [39]. Elevated expression of the IGF2 gene is also associated with high-grade, advanced stage ovarian cancer and is predictive of poor survival [40]. Furthermore, dysregulation of IGF1R is found in many cancers [41]–[45] including ovarian cancer, where overexpression of IGF1R correlates with poor prognosis [46]. Finally, the levels of IGFBPs vary between healthy and diseased states; for example, IGFBP3 is the most abundant IGFBP in serum and its levels are inversely correlated with risk of developing high-grade advanced stage ovarian cancer [47]–[49]. Combined, these studies suggest that changes that increase the potential for IGF1-IGF1R interaction (*i.e.,* increased IGF1/IGF1R, decreased IGFBPs) promote ovarian cancer and that the IGF network is a promising therapeutic target.

Therapeutically, the IGF network has been targeted by three distinct mechanisms: tyrosine kinase inhibitors against IGF1R, monoclonal antibodies to prevent ligand binding to IGF1R, and neutralizing antibodies against IGF1 and/or IGF2 [50]. Due to the similarity between IGF1R and IR, tyrosine kinase inhibitors against this network can lead to side effects such as elevated blood glucose and insulin levels [51],[52]. Antibodies against the IGF1R are more specific, but still have the potential to interfere with IGF1R/IR heterodimers, leading to off-target effects. Therefore, the most specific way to interfere with IGF signaling is through the use of ligand-neutralizing antibodies. Trials with members of all three classes are ongoing in several tumor types. A phase I trial of figitumumab, a monoclonal antibody against IGF1R, reported that therapy was well tolerated in combination with chemotherapy, and a complete response was observed in the ovarian cancer patient that was enrolled [53]. Similar to many molecularly-targeted therapies, results from clinical trials that target the IGF network suggest that these inhibitors will not have broad efficacy and will instead work best when provided to a subset of patients [2],[50],[54]. However, it remains difficult to predict how tumor cells will respond to IGF ligands or IGF-targeted inhibitors as the IGF system is a complex network with many different players. For example, preclinical studies with figitumumab suggested that elevated IGF1R levels were predictive of response [55] while analysis of responses in the phase I trial suggested that patients with a high baseline IGF1:IGFBP3 ratio were more likely to respond [53].

To better apply IGF-targeted therapies, it will be essential to move beyond the qualitative understanding of the role of IGF ligand, receptor, and binding protein levels and systematically analyze this network. Therefore, to examine the hypothesis that a quantitative balance between the levels of different components of the IGF system (*i.e.,* IGF1, IGFBPs, and IGF1R) determines cellular response and impacts sensitivity to anti-IGF therapies, we experimentally examined ovarian cancer cell proliferation and cellular mechanisms that regulate IGF1 availability. We then developed a mass-action model to analyze how the interactions between these components impacted the steady-state level of IGF1-IGF1R complexes, which initiate downstream signaling to impact cell behavior. Using this model, we predicted and experimentally confirmed how changes in the levels of IGFBPs impact cell proliferation and examined the efficacy of IGF1R-blocking and IGF1-neutralizing antibodies against IGF networks with varying levels of IGF1, IGF1R, and IGFBPs.

## Results and discussion

### Proliferation in response to IGF1 was dose, and not concentration, dependent

**Figure 1:**
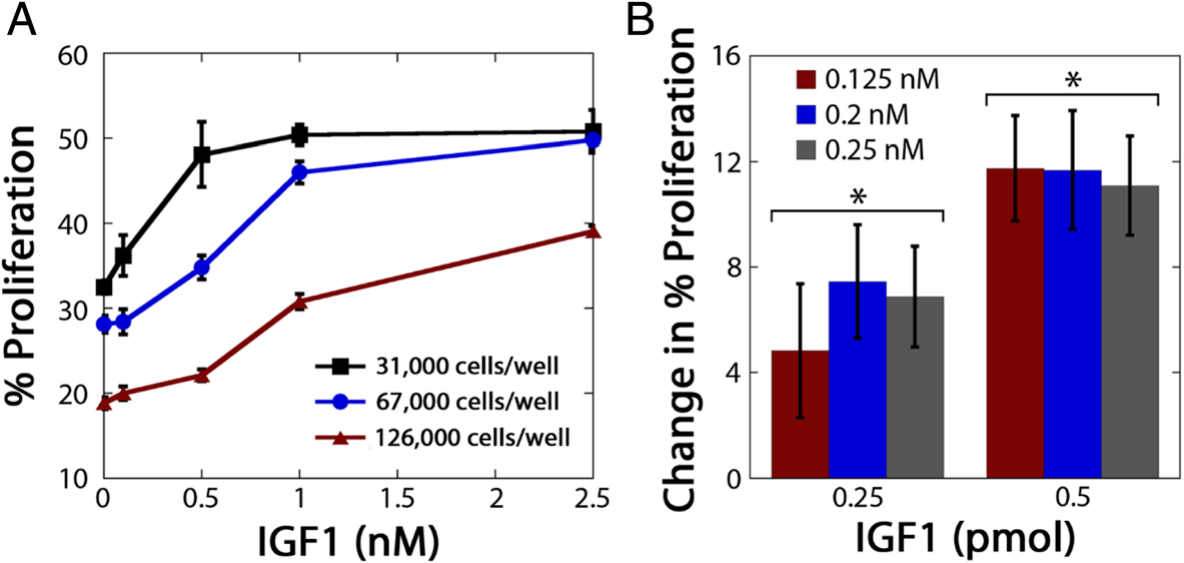
**OVCAR5 proliferation was dependent on both cell density and IGF1 dose.A**, OVCAR5 exhibited concentration-dependent proliferation in response to IGF1 treatment at all three cell densities (31,000, 67,000, 126,000 cells/well); however, the extent of proliferation induced by a set concentration of IGF1 treatment was different at the three cell densities. **B**, Treatment dose (*i.e.,* pmol of IGF1) impacted the extent of OVCAR5 proliferation while concentration had minimal effect. OVCAR5 were plated at a fixed density (116,000 cells/well) to control for cell confluency, and treatment volumes were varied to result in two doses of IGF1 at three different concentrations. *indicates significantly different (p < 0.05) between doses for each concentration, n = 3 per treatment.

While OVCAR5 cells have previously been reported to proliferate in response to treatment with IGF1 [56], there are no reports describing how these cells respond to varying levels of IGF1 that would allow us to begin addressing the hypothesis that a quantitative balance between receptor, ligand, and binding proteins controls cell response. Therefore, we first characterized the response of OVCAR5 cells to a range of physiologically-relevant IGF1 concentrations [57]–[59]. When OVCAR5 cells were treated with increasing concentrations of IGF1, cells were observed to proliferate in a concentration-dependent manner (Figure 1A). Interestingly, this relationship was dependent upon the cell confluency at the time of treatment, with OVCAR5 exhibiting a more robust increase in proliferation for a given concentration of IGF1 when cells were plated at a lower cell density. As the number of cells increases, there will be a decrease in the dose (*i.e.*, mass) of IGF1 that each cell receives for a given concentration, potentially explaining the observed decrease in sensitivity at higher cell densities. The concentration where IGF1-induced proliferation saturated was also dependent on cell density, with saturation at concentrations as low as 0.5 nM IGF1 for the lowest cell density, whereas for the highest cell density tested saturation was not observed. This is consistent with the potential importance of considering the balance between IGF1 and IGF1R levels; for higher cell densities, it would take a larger dose of IGF1 to saturate the available IGF1R pool. Importantly, the baseline proliferation of cells that were vehicle-treated was also related to cell density, with higher proliferation rates for cells at lower densities. This observed difference in baseline proliferation at different cell densities is likely due to density-dependent contact inhibition of cell proliferation [60],[61].

To control for the effect of contact inhibition and examine if the observed differences were a result of variations in the levels of different IGF system components (*i.e.,* IGF1, IGFBPs, and IGF1R), we next examined if cell response was dependent on the IGF1 dose, rather than IGF1 concentration, at a fixed density. OVCAR5 were plated at a fixed density and treated with two different doses of IGF1 (0.25 or 0.5 pmol) at three different concentrations (0.125 – 0.25 nM) by varying the volume of cell culture media. As expected, the level of induced proliferation increased with increasing IGF1 dose (Figure 1B). Importantly, this effect was truly dose-dependent rather than concentration-dependent, as within each dose increasing concentration did not have a significant effect. Experiments with vehicle-treated cells confirmed that the different volumes of cell culture media did not impact baseline proliferation (Additional file 1). Additionally, the selected concentrations were below the concentrations that resulted in saturation in the initial experiments (Figure 1A), such that the lack of concentration-dependence was not a result of saturation. One potential limitation of this interpretation is the relatively small dose range selected. Unfortunately, due to limitations in well depth it was not possible to test a broader range of conditions in standard tissue culture setups.

These results demonstrate that cell response to IGF1 is dependent on the dose of IGF1 that is available per cell, whether that ratio is altered by cell density (Figure 1A) or changes in the amount of ligand provided, independent of concentration (Figure 1B). The principle that cells respond to the total dose and not concentration has been demonstrated in other growth factor signaling networks. For example, the potency of a given concentration of transforming growth factor-β (TGF-β) on intracellular Smad signaling depended on the number of cells or media volume, and was more accurately described when considered in terms of TGF-β molecules/cell and not bulk concentration [62]. This interpretation that concentration is not the best predictor of cell response may seem surprising as isolated receptor-ligand binding equilibrium in *in vitro* assays are governed by concentration-dependent kinetics. However, in intact cellular experiments, the actual concentration of ligand available for each receptor is dependent on multiple factors such as cell number (which alters receptor number) and media volume (which impacts the total amount of ligand, and therefore, ligand depletion kinetics). As a consequence, cell response for growth factor systems may be more consistent if characterized in terms of the ligand dose per cell instead of bulk concentration. These findings have important ramifications for experimental design and interpretation. For example, researchers frequently conduct experiments in several different size plates and commonly apply the same concentration of ligand across these plates. However, if the cell number and media volume are not considered, this will likely result in applying different doses of ligand per cell across the different experiments, which may lead to experimental inconsistencies. In our results using IGF1, the impact of cell density was not as prominent at higher doses similar to those used in many prior experiments with the IGF system [63],[64]; however, studies that are conducted at physiologically relevant concentrations around 1 nM appear likely to be impacted by these variations [57]–[59]. Given recent concerns about the reproducibility of key findings in cancer research [65], metrics such as cellular dose that may better enable experimental consistency should be utilized.

### IGF1 was depleted by both intracellular and extracellular mechanisms

**Figure 2:**
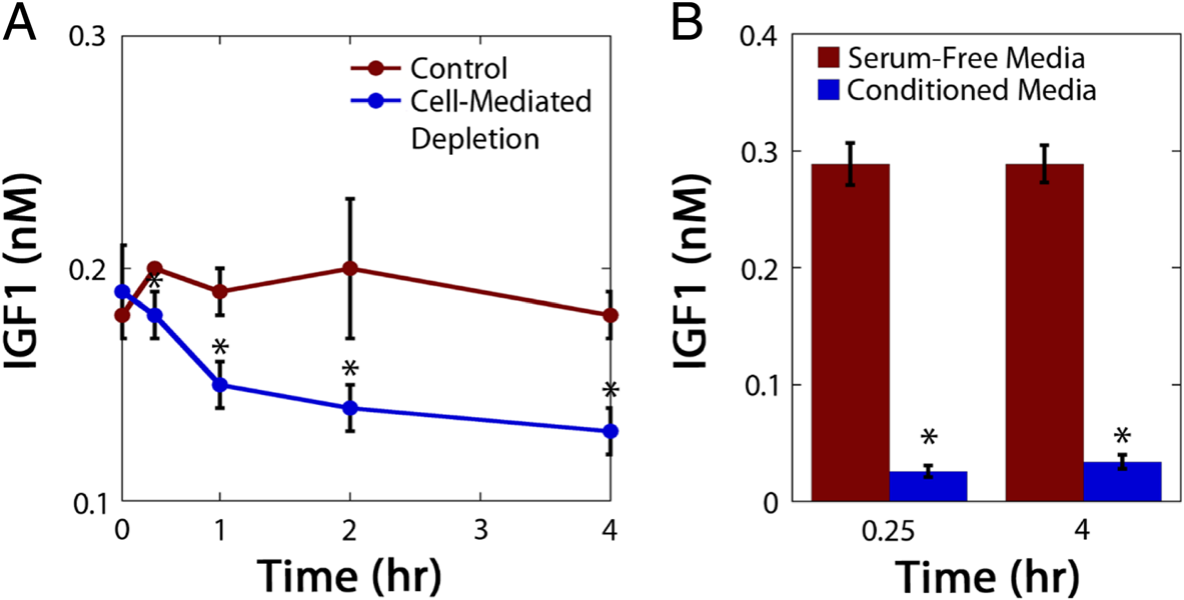
**IGF1 availability was regulated by cell-mediated ligand depletion and IGFBP sequestration.A**, IGF1 was depleted by OVCAR5 in the absence of IGFBPs. **B**, The majority of IGF1 added to conditioned media was sequestered by cell-secreted IGFBPs. *indicates significant difference (p < 0.05) from cell-free control for **A** or from serum-free media control for **B**, n = 3 per treatment.

As cell proliferation in response to IGF1 was dependent upon the dose of IGF1 available for each cell, the mechanisms that regulate the level of free extracellular IGF1 would be expected to impact cell response. One likely mechanism of IGF1 depletion from the extracellular environment is receptor-mediated endocytosis of IGF1 [66],[67], via both caveolin- and clathrin-mediated pathways [21],[68]. To determine if OVCAR5 depleted IGF1 from cell culture media, cells were plated at a fixed density (as in Figure 1B; this density was used for all remaining experiments), changed to fresh serum-free media to remove accumulated IGFBPs, treated with IGF1, and the depletion of IGF1 from cell culture media was measured over time by ELISA (Figure 2A). The amount of free IGF1 present in the cell culture media decreased over time, suggesting that OVCAR5 depleted IGF1 through receptor-mediated endocytosis. To confirm that the observed depletion in Figure 2A was the effect of cell-mediated endocytosis and not the result of newly-produced IGFBPs sequestering IGF1, this experiment was also performed with OVCAR5 treated with the protein synthesis inhibitor cycloheximide, to prevent the production and accumulation of secreted IGFBPs (Additional file 2). From Additional file 2, the sequestration of IGF1 by secreted IGFBPs was not significant until after 4 hours, strongly suggesting that the observed depletion in Figure 2A was the result of cell-mediated endocytosis. In other receptor systems, ligand depletion by endocytosis has been shown to have significant effects on cell behavior. For example, endocytosis of ligand-activated epidermal growth factor receptor (EGFR) was required for signal attenuation [69]. Additionally, variation in ligand depletion rate was recognized as a mechanism behind the difference in mitogenic potency of transforming growth factor-α (TGF-α) and EGF. While TGF-α and EGF both signal through the EGF receptor, TGF-α was depleted much faster from the extracellular environment and as a result was a weaker stimulus compared to EGF [70]. Finally, ligand depletion appears to be critical in the TGF-β network as the potency of a set TGF-β dose depended upon the number of cells to which it was applied and the duration of Smad activity correlated to the duration of time that TGF-β was present [62].

In addition to cell-mediated endocytosis, the IGF system *in vivo* has another layer of regulation to modulate extracellular levels of IGF1, the IGFBPs [27]. To determine if OVCAR5 secrete IGFBPs into the extracellular environment *in vitro* and quantify the subsequent IGF1 sequestration by these IGFBPs, we utilized an IGF1 ELISA that specifically detects free IGF1 in cell culture media to compare the amount of IGF1 in serum-free media versus OVCAR5-conditioned media (Figure 2B). The sequestration of free IGF1 in the conditioned media was rapid, occurring within 15 minutes, and stable for at least 4 hours. These results confirmed that OVCAR5 secreted IGFBPs into the media and that up to 90% of IGF1 applied was sequestered by these cell-secreted IGFBPs, resulting in an actual treatment dose that was substantially less than the applied dose. The observed depletion was much more significant than in the IGFBP-free scenario described above (Figure 2A), indicating that IGF1 sequestration by IGFBPs was the predominant mode regulating IGF1 levels for OVCAR5 cells. As demonstrated in Figure 1B, the actual amount of IGF1 impacts cell proliferation response; therefore, accounting for the depletion of IGF1 through IGFBP sequestration may be necessary to accurately predict cell proliferation.

Combined with previous reports, our results indicate that mechanisms that regulate extracellular ligand levels may be a universal control element of receptor systems [62],[69],[70]. The impact of these mechanisms is especially important in high-throughput screens such as microfluidic research platforms where the volume of media for each cell is reduced and application of the same concentrations as in bulk experiments may result in a substantially lower cellular dose, which would be more quickly depleted. Importantly, IGFBP sequestration may lead to different effects on cellular response than receptor-mediated degradation, as IGFBPs can protect IGF1 from degradation and alter activity [24]. Therefore, it will be important to develop a more detailed understanding of how IGFBP sequestration impacts cell response to understand ovarian cancer cell responses to IGF1 and determine how to utilize the processes that govern ligand availability to control cell behavior, both experimentally and potentially therapeutically.

### Steady-state levels of IGF1-IGF1R complexes predicted cellular response

**Figure 3:**
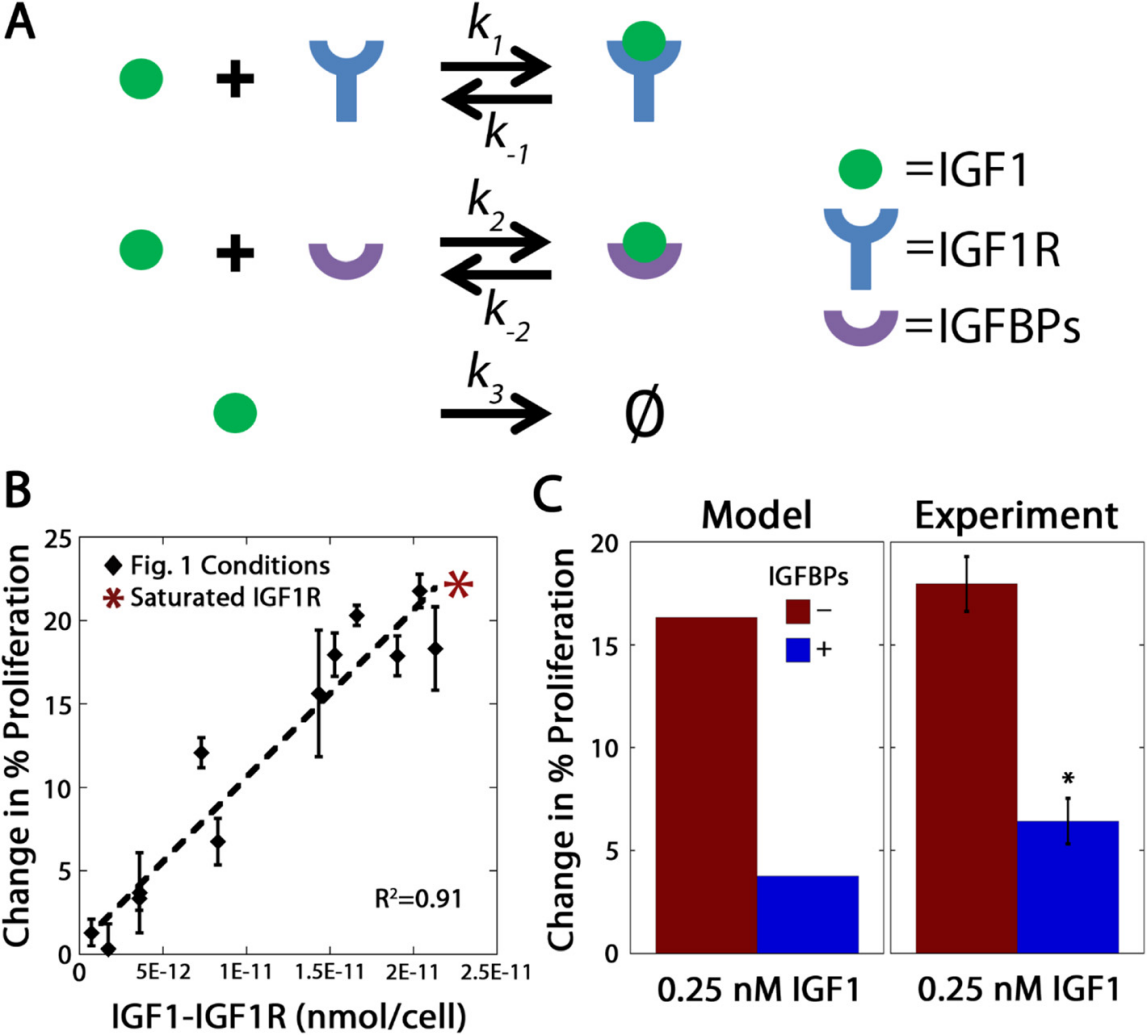
**IGF1-induced proliferation was a function of steady-state levels of IGF1-IGF1R complexes.A**, Diagram of interactions included in the model. **B**, The computationally-determined concentration of steady-state levels of IGF1-IGF1R complexes exhibited a linear relationship with the experimentally-observed increase in proliferation between IGF1-treated OVCAR5 and vehicle controls. Theoretical saturation of IGF1R is represented by an *. **C**, Model predictions and experimental results of the effect of IGFBPs on OVCAR5 proliferation in response to IGF1 treatment. The steady-state model predicted that the presence of IGFBPs in the cell culture media would reduce steady-state levels of IGF1-IGF1R complexes and result in decreased cell proliferation. Experimental tests confirmed both the qualitative and quantitative extent of this IGFBP effect. *indicates significant difference (p < 0.05) from IGFBP-negative condition, n = 3 per treatment.

Combined, these results indicated that IGFBPs and IGF1R regulate IGF1 level in the extracellular environment. As the level of IGFBPs and IGF1R scale with cell number, this can qualitatively explain the observed differences in sensitivity to IGF1 at different cell densities. To study the balance of these components quantitatively, we developed the first model of the IGF network in ovarian cancer using mass-action kinetics to examine these interactions in more detail. The model was developed to analyze the binding interactions between IGF1 with IGFBPs and IGF1R, assuming reversible interactions between IGF1 and IGFBPs, and between IGF1 and IGF1R (Figure 3A). Initial conditions and rate coefficient values used in the model are provided in Table 1. The principal output of this model is the level of IGF1-IGF1R complexes at steady-state for given initial levels of IGF1, IGF1R, and IGFBPs. This model was used to calculate the level of IGF1-IGF1R complexes per cell at steady-state for each of the experimental conditions presented in Figure 1A. When the model calculated level of IGF1-IGF1R complexes per cell was compared to the extent of proliferation induced by IGF1 (Figure 3B), we observed a linear relationship where increasing levels of IGF1-IGF1R complexes correlated with increased proliferation. Interestingly, as the level of IGF1-IGF1R increased the experimentally-observed change in proliferation saturated. This suggests that there is a maximum proliferation response corresponding to the occupation of every available IGF1R per cell, beyond which additional treatment with IGF1 will result in no further change in cell proliferation. To test this interpretation, we utilized the model to determine the maximum level of IGF1-IGF1R complexes per cell, corresponding to occupation of every IGF1R. As seen in Figure 3B, the predicted level of proliferation for this maximum was comparable to the observed saturation. Our results suggest that OVCAR5 proliferation depends upon receptor occupancy (*i.e.,* the total number of receptor-ligand complexes per cell) and not solely on the level of IGF1. Interestingly, a similar linear relationship has been reported for the level of steady-state EGF receptor occupancy and DNA synthesis rate, demonstrating that relatively simple mathematical models can explain complex biological phenomena [71],[72].

**Table 1:** Initial conditions and rate coefficient values

**Initial condition**	**Value**
IGFBPs per cell^a^	1.21 × 10^−8^ nmol/cell
IGF1R per cell^a^	2.23 × 10^−11^ nmol/cell
Reference cell number *N*_*0*_^a^	116,000 cells/well
**Rate coefficient**	**Value**
Association rate coefficient of IGF1-IGF1R complex (*k*_*1*_)^b^	1 nM^−1^ hr^−1^
Dissociation rate coefficient of IGF1 and IGF1R (*k*_*−1*_)^b^	1 hr^−1^
Association rate coefficient of IGF1-IGFBP complex (*k*_*2*_)^b^	1 nM^−1^ hr^−1^
Dissociation rate coefficient of IGF1 and IGFBP (*k*_*−2*_)^b^	0.1 hr^−1^
Cell-mediated IGF1 depletion rate coefficient (*k*_*3,0*_)^a^	0.017 hr^−1^

^a^Experimentally determined for OVCAR5 cells.

^b^Based on *K*_*d*_*=* 1 nM for IGF1 with IGF1R and *K*_*d*_*=* 0.1 nM for IGF1 with IGFBPs [24],[26],[87]–[94].

A key advantage of developing computational models is that they can be easily used to predict the effects of different perturbations to the system. As a test of our model’s predictive ability, we examined the effect of changes in the level of IGFBPs, which impact the level of free IGF1 (Figure 2B), on OVCAR5 sensitivity to IGF1. To predict the effect of this IGF1 sequestration on cell proliferation, model equations were solved for two different scenarios, one corresponding to OVCAR5-conditioned media containing cell-secreted IGFBPs and one corresponding to fresh serum-free media in which no IGFBPs were present. The resulting model predictions of the steady-state level of IGF1-IGF1R complexes were used in conjunction with the linear relationship depicted in Figure 3B to predict the cell proliferation response for these two experimental conditions. The model predicted that in the absence of IGFBPs, more IGF1 would be free to form IGF1-IGF1R complexes and consequently, IGF1 treatment would elicit more proliferation. To experimentally validate this model prediction, OVCAR5 proliferation was measured in conditions that were positive or negative for IGFBPs by spiking the IGF1 treatment into OVCAR5-conditioned media or serum-free media, respectively. As seen in Figure 3C, the model predictions demonstrated qualitative agreement with experimental measurements, with more proliferation induced in the IGFBP-negative condition compared to the IGFBP-positive condition. Additionally, the model prediction and experimental results were in close quantitative agreement, with a less than 2% difference. These results provide support for the model’s ability to predict proliferation from the steady-state levels of IGF1-IGF1R complexes and suggest that quantitative analysis of the balance between components in the IGF network may help to elucidate mechanisms regulating cellular responses.

Interestingly, our experimental validation further demonstrates that experimental analysis of cellular sensitivity to IGF1 can be dramatically impacted by the specifics of the experimental protocol. When IGF1 treatment was spiked into OVCAR5-conditioned media, the amount of free IGF1 was lower than the applied concentration as a result of IGFBP sequestration and IGF1-mediated proliferation was subsequently decreased. In contrast, when IGF1 treatment was added to fresh serum-free media by changing the cell culture media, there were no IGFBPs present to sequester IGF1 during early times (Additional file 2) and as a result, IGF1-mediated proliferation was significantly increased (Figure 3C). The method used to apply ligand is rarely specified in experimental protocols, providing another potential source of experimental inconsistency. This factor may also impact other growth factor networks that do not have binding proteins, through the accumulation of cell-secreted proteases that impact ligand stability [73].

### Model analysis of IGF1-neutralizing and IGF1R-blocking antibodies

**Figure 4:**
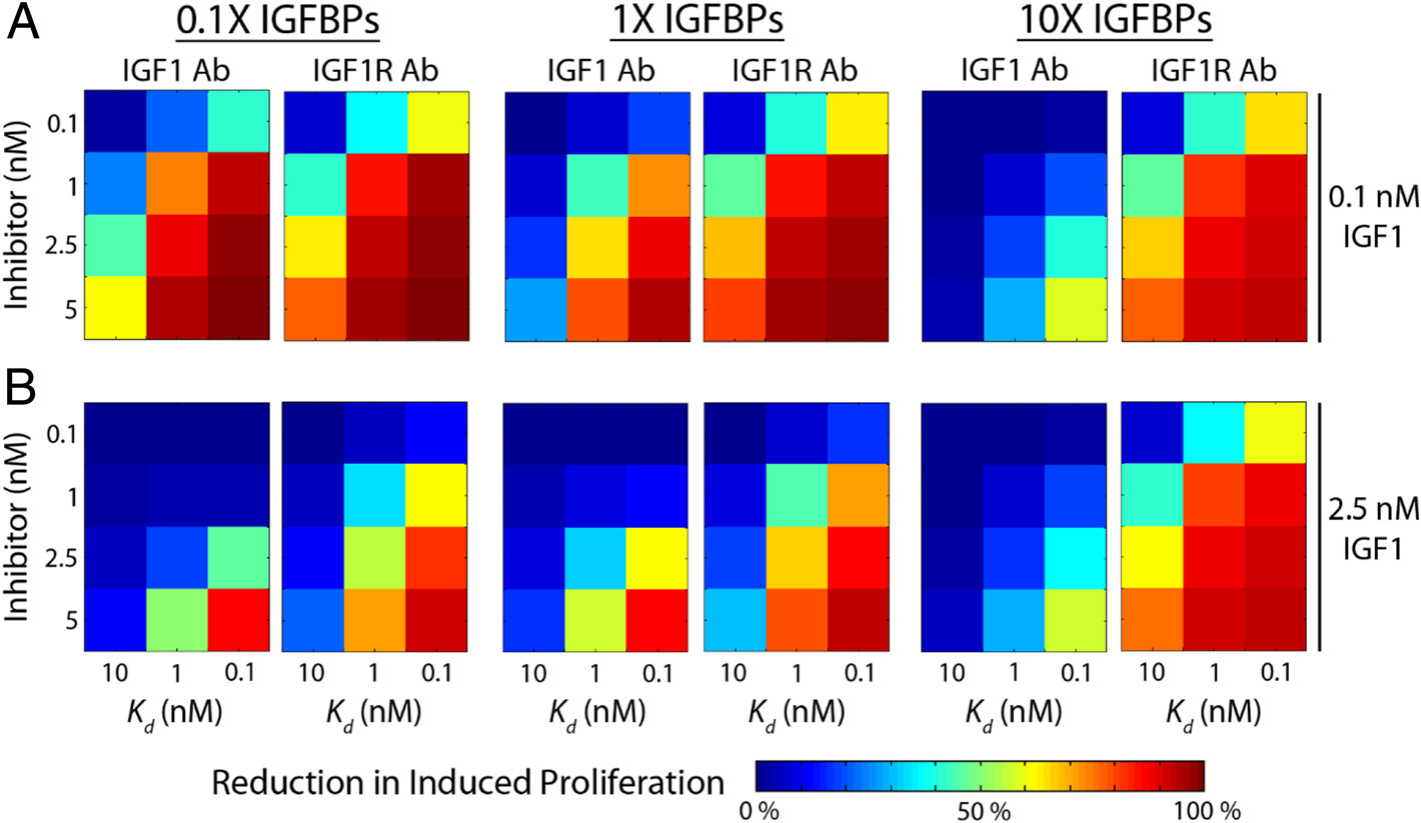
**Model-predicted reduction in cell proliferation in response to antibody treatment indicated that IGF1R-blocking antibodies will be more effective than IGF1-neutralizing antibodies.** A range of antibody dissociation constants (*K*_*d*_, 0.1-10 nM) were used to simulate the effect of high to low binding affinity. The effects of the antibody in the presence of three different IGFBP concentrations at **A**, low (0.1 nM) or **B**, high (2.5 nM) IGF1 level were determined using the steady-state model. Model results indicated that an antibody that blocks IGF1R would more strongly decrease the steady-state concentration of IGF1-IGF1R complexes and consequently, inhibit IGF1-induced cell proliferation, than an antibody that binds and neutralizes IGF1.

Given that our model can accurately predict the effects of perturbations to the network, we next used it to analyze the impact of different therapeutic options. This analysis is particularly relevant for anti-IGF therapy as there are multiple approaches in clinical trials and results from these trials suggest that variability in the levels of different IGF system components between patients may impact efficacy [53],[55]. The IGF system can be targeted specifically through antibodies that bind IGF1 to neutralize its activity or through antibodies that bind to IGF1R to block ligand binding [50]. Our model analysis demonstrated that IGF1 sequestration via IGFBPs was a viable means to decrease the level of IGF1-IGF1R complexes and inhibit cell proliferation (Figure 3C); therefore, an antibody that neutralizes IGF1 could conceivably be the more effective avenue to halt IGF1-mediated cell proliferation. To compare these two strategies we modified the model to include the different antibody types using a range of dissociation constants (*K*_*d*_) and doses. To examine how these therapies were impacted by variation in the IGF network levels, the antibodies were tested against several variations in the level of IGF1, IGF1R, and IGFBPs to determine the impact on IGF1-induced proliferation (Figure 4).

The model predicted that treatment with the IGF1R-blocking antibody will have a stronger absolute effect on cell proliferation than the IGF1-neutralizing antibody at low and moderate antibody doses, and that both types of antibodies will significantly reduce cell proliferation for high antibody doses. Predictably, the effect of both antibody types was more pronounced for conditions of low IGF1 dose than for high IGF1 dose, and in the limit of low IGF1 dose and a low level of IGFBPs the effects of both types of antibodies were similar. However, in these conditions the extent of IGF1-induced proliferation was already modest (Figure 3B). In contrast, the difference in effectiveness between the two antibody types was more pronounced under conditions of high IGFBP levels, where the IGF1-neutralizing antibody had relatively little effect while the effect of the IGF1R-blocking antibody was significantly enhanced. The reduction in the efficacy in the IGF1-neutralizing antibody with increasing IGFBP levels arises from the direct competition between IGFBPs and IGF1-neutralizing antibody for free IGF1 in solution. Meanwhile, the effectiveness of the IGF1R-blocking antibody is largely determined by the relative difference between the levels of IGF1 and IGF1R-blocking antibody. High levels of IGFBPs sequester large amounts of IGF1, effectively reducing the level of IGF1 and actually enhance the impact of IGF1R-blocking antibody relative to low IGFBP conditions. Thus, while the model results demonstrated that sequestration of IGF1 by IGFBPs or by an IGF1-neutralizing antibody inhibits cell proliferation, an antibody which blocks IGF1R is predicted to be the more effective tool for impeding IGF1-mediated cell proliferation. To further confirm the effectiveness of an IGF1-neutralizing antibody to an IGF1R-blocking antibody, we directly analyzed the relative inhibition of IGF1-neutralizing antibody compared to IGF1R-blocking antibody (Additional file 3). In this analysis a ratio greater than 1 indicates that the IGF1-neutralizing antibody would have a stronger effect and a ratio less than 1 indicates that the IGF1R-blocking antibody would be more effective. In all scenarios examined, this ratio was less than 1 and the IGF1R-blocking antibody would be predicted to be a more effective method. This conclusion remains robust over a wide range of IGF1R levels as increasing the initial receptor level by a factor of 10-fold had virtually no impact on this interpretation (Additional file 4). This arises from the fact that for even relatively low doses of IGF1, the level of IGF1-IGF1R complexes is most strongly limited by the level of available IGF1R.

Importantly, our model predictions of the efficacy of an IGF1-neutralizing or IGF1R-blocking antibody were extrapolated from experimental data collected *in vitro* and would need further validation to conclusively predict *in vivo* behavior, particularly for long-term treatment that may result in receptor down-regulation [74],[75]. While the present model constitutes an essential foundation, inclusion of all receptor and ligand components of the IGF network will be necessary to develop a comprehensive framework for modeling downstream signaling pathways in order to obtain a complete understanding of the IGF system in ovarian cancer development and progression. For example, a limitation of our current model is the exclusion of signaling competent IGF1R/IR heterodimers, which can induce cell proliferation [76]. Heterodimers were neglected in the present model because the cell line utilized in this study exhibited an insignificant heterodimer population as a fraction of total receptors (Additional file 5) and inhibition of IR activity did not decrease IGF1-induced proliferation (Additional file 6); however, a more broadly applicable model may need to include their effect. Heterodimers of IGF1R/IR are preferentially activated by IGF ligands; therefore, treatment with anti-IGF therapies would also impact IGF1R/IR receptor activity. For example, ganitumab (AMG 479), a monoclonal antibody against IGF1R, has been shown to be effective against inhibiting IGF ligand stimulated activation of IGF1R/IR heterodimers [77],[78].

Importantly, expansion and refinement of foundational models similar to the one developed in this system has yielded fruitful understanding of the EGF system [79]–[82]; similarly, we anticipate that expanding upon the model developed in this study will lead to further insights into the role of the IGF system in ovarian cancer. For example, a model of IGF1R signaling in glial cells suggested that IGF1R internalization and recycling was essential for extended phosphorylation of AKT [21] and a model of IGF1R signaling in breast cancer cells identified optimal drug combinations to inhibit signaling [83]. Importantly, neither of these models examined the impact of the IGFBPs. Recently, a network of IGF1, IGF2, receptors, and binding proteins was modeled to examine how these interactions regulate the distribution of IGF1-IGF1R complexes in articular cartilage [84]. While this study did not examine how IGF1-IGF1R levels influenced cellular behavior, this more complex model also suggested IGFBP levels were key in regulating receptor-ligand complex levels. Inclusion of the additional receptor and ligand components of the IGF network will be essential to develop a framework for modeling downstream signaling pathways in order to obtain a more complete understanding of the IGF system in ovarian cancer development and progression.

## Conclusions

Though the IGF system is a promising therapeutic target, the principles regulating ovarian cancer cell response to IGF ligands have not been systematically studied and it is difficult to predict how cells will respond to IGF ligands or IGF inhibitors. In this study, we determined that cell response to IGF1 treatment can be better predicted in terms of the absolute amount of IGF1 rather than the applied concentration, suggesting that experimental tests with IGF ligands should be described in units of ligand dose per cell rather than standard concentrations. As cell proliferation in response to IGF1 was dependent upon the total dose of IGF1, we examined the mechanisms that regulate the amount of free IGF1 and determined that cell-secreted IGFBPs in the extracellular environment were the primary mechanism to regulate IGF1 levels. To further understand the principles that govern IGF1-mediated proliferation, a mass-action model was developed to study the binding interactions of IGF1 with IGFBPs and IGF1R, and model analysis demonstrated that the steady-state level of IGF1-IGF1R correlated to IGF1-induced proliferation and that changes in the levels of IGFBPs had predictable effects on proliferation. The suppression of cell proliferation through antibody treatment has received considerable focus as a means of combatting cancer. However, it is not clear which component of the IGF system is the most promising target for antibody treatment. To gain fundamental insight into the impact of targeted antibody treatment on IGF-mediated cell proliferation, the model was utilized to examine the effects of treating with an antibody that either neutralizes IGF1 or blocks IGF1-IGF1R binding on IGF1-induced proliferation. The model predicted that an IGF1R-blocking antibody would be more effective at inhibiting proliferation than an IGF1-neutralizing antibody, mainly due to the fact that the level of IGF1-IGF1R complexes was receptor limited, and that this effect would be even more pronounced under conditions of high IGFBP concentrations. Future modeling work will build upon the model developed here, in the continued effort to identify clinically-relevant drug targets or determine how levels of different components of growth factor systems influence sensitivity to therapies [85].

## Methods

### Reagents and cell culture

All reagents were from Sigma-Aldrich (St. Louis, MO) unless otherwise noted. OVCAR5 cells, an immortalized cell line originally isolated from a patient with serous ovarian cancer, were obtained from Dr. R. Bast (MD Anderson Cancer Center, Houston, TX) and are a member of the NCI-60 panel of cell lines. Cells were maintained at 37°C in a humidified 5% CO_2_ atmosphere in a complete culture medium composed of 1:1 (v/v) ratio of MCDB 105 and Medium 199 (Corning, Manassas, VA) supplemented with 10% fetal bovine serum (Life Technologies, Carlsbad, CA) and 1% penicillin/streptomycin. OVCAR5 cells were routinely tested and confirmed to be mycoplasma negative using the MycoAlert® Mycoplasma Detection Kit (Lonza, Rockland, ME).

### Ethical approval

Studies were performed using a publicly-available immortalized cell line (OVCAR5) without any identifiable information; therefore, the studies are not subject to humans subject review.

### Quantification of cell proliferation

OVCAR5 proliferation in response to IGF1 was measured under a variety of conditions. First, OVCAR5 were seeded in 12-well plates at different densities (5,000, 10,000, or 20,000 cells/well), allowed to grow for 2 days, and then serum-starved for 24 hours (resulting in final densities of 31,000, 67,000, and 126,000 cells/well, respectively) prior to treatment with exogenous recombinant human IGF1 (Peprotech, Rocky Hill, NJ). In select experiments, a constant cell confluency was achieved at the time of IGF1 treatment by seeding OVCAR5 in 12-well plates at 77,740 cells/well, allowing cells to attach for 6 hours, and then serum-starving for 24 hours prior to treatment with IGF1 (a final density of 116,000 cells/well). For these experiments, IGF1 was spiked directly into the serum-free media that cells had been cultured in, which may contain cell-secreted IGFBPs. To measure OVCAR5 proliferation in response to IGF1 treatment in the absence of IGFBPs, the serum-free media was aspirated, cells were rinsed once with PBS, and the IGF1 treatment was added with fresh serum-free media. IGF1 treatment units discussed in this paper are provided as either dose (pmol, the total amount of ligand added) or concentration (nM). All experiments were done with 1 mL of media per well. Cell proliferation was quantified after 24 hours of IGF1 treatment using the Click-iT® EdU Alexa Fluor® 488 flow cytometry assay (Life Technologies) according to manufacturer’s instructions. Cells were incubated with EdU for 6 hours prior to sample collection and analyzed on a BD Accuri™ C6 flow cytometer (BD, Franklin Lakes, NJ). Samples were gated for the EdU-positive population, which is a measure of the percentage of S-phase cells, to determine the proliferation percentage.

### Quantification of ligand depletion

Two mechanisms to modulate the extracellular concentration of IGF1 were examined: cell-mediated depletion of ligand and extracellular sequestration by IGFBPs. To measure cell-mediated IGF1 depletion, OVCAR5 were seeded in 12-well plates at 77,740 cells/well, allowed to attach for 6 hours, and then serum-starved for 24 hours. Prior to IGF1 treatment, the media was aspirated, cells were rinsed once with PBS, and fresh serum-free media was added to the cells to ensure minimal levels of IGFBPs were present during IGF1 treatment. Over a period of 4 hours of IGF1 treatment, cell culture media was collected from each sample, briefly centrifuged at 200 g for 10 min at 4°C to remove cellular debris, and the amount of IGF1 remaining in the culture media was determined by the IGF1 ELISA (R&D Systems, Minneapolis, MN). To control for IGF1 adsorption to tissue culture plastic, controls were collected in the same manner from wells that did not have OVCAR5 seeded in them. To quantify IGF1 sequestration by cell-secreted IGFBPs, 0.25 nM IGF1 was spiked into fresh serum-free media or conditioned media collected after 24 hours of culture with OVCAR5 cells plated as described above. The amount of free IGF1 in each condition was determined by the same IGF1 ELISA, which is specific for IGF1 that is not sequestered by IGFBPs. ELISAs were performed according to manufacturer’s instructions using a Tecan Infinite® M1000 plate reader (Tecan Group Ltd., Switzerland).

### Mass-action model of IGF1 network

A mass-action kinetics model was developed to analyze the binding interactions between IGF1 with IGFBPs and IGF1R. The mathematical model focused on IGF1 interactions with IGFBPs and IGF1R and did not include IGF2R or IR as IGF1 cannot be bound by IGF2R [86] and IGF1-induced proliferation was determined to be independent of IR kinase activity (see Additional file 6). This model is described by the following system of ordinary differential equations:

(1) $$\frac{dC_{1}}{\mathrm{dt}}=k_{-1}C_{1:1R}+k_{-2}C_{1:\mathrm{BP}}-k_{1}C_{1}C_{1R}-k_{2}C_{1}C_{\mathrm{BP}}-k_{3}C_{1}$$

(2) $$\frac{dC_{1R}}{\mathrm{dt}}=k_{-1}C_{1:1R}-k_{1}C_{1}C_{1R}$$

(3) $$\frac{dC_{1:1R}}{\mathrm{dt}}=k_{1}C_{1}C_{1R}-k_{-1}C_{1:1R}$$

(4) $$\frac{dC_{\mathrm{BP}}}{\mathrm{dt}}=k_{-2}C_{1:\mathrm{BP}}-k_{2}C_{1}C_{\mathrm{BP}}$$

(5) $$\frac{dC_{1:\mathrm{BP}}}{\mathrm{dt}}=k_{2}C_{1}C_{\mathrm{BP}}-k_{-2}C_{1:\mathrm{BP}}$$

where *C*_*i*_ is the concentration of component *i* and the subscripts *1*, *1R*, and *BP* refer to IGF1, IGF1R, and IGFBP, respectively. For these reactions, *k*_*1*_ is the association rate coefficient of IGF1-IGF1R complex, *k*_*−1*_ is the dissociation rate coefficient of IGF1 and IGF1R, *k*_*2*_ is the association rate coefficient of IGF1-IGFBP complex, and *k*_*−2*_ is the dissociation rate coefficient of IGF1 and IGFBP. *k*_*3*_ is the cell-mediated IGF1 depletion rate coefficient and was assumed to be proportional to cell number according to the equation:

(6) $$k_{3}=k_{3,0}\left[N/N_{0}\right]$$

where *N* is the number of cells, *N*_*0*_ is a reference cell number, and *k*_*3,0*_ is the value of *k*_*3*_ measured at the reference cell number *N*_*0*_. The value of *k*_*3,0*_ was determined to be 0.017 hr^−1^, by half-life analysis of the IGF1 concentration data depicted in Figure 2A for reference cell number *N*_*0*_ of 116,000 cells/well. The model assumes reversible interactions between IGF1 and IGFBPs, and between IGF1 and IGF1R. The binding affinity of all six structurally related IGFBPs for IGF1 are reported to be within the same order of magnitude [24]; therefore, for model simplification IGFBP1-6 were consolidated into one term. While IGFBPs under certain conditions can potentiate IGF action, we assumed that the sole action of IGFBPs *in vitro* was to sequester IGF1 from binding to IGF1R. The reaction rate coefficients were determined using published binding affinity values for the binding of IGF1 with IGFBPs (*K*_*d*_ = 0.1 nM) and the binding of IGF1 with IGF1R (*K*_*d*_ = 1 nM) that were measured in intact cells rather than from isolated receptors, in order to better mimic the experimental setup [24],[26],[87]–[94]. The timescale of the binding and unbinding interactions of IGF1 with IGFBPs and IGF1R is expected to be much shorter than the timescale of cell proliferation. Therefore, the kinetics were assumed to be sufficiently fast that the system can reach steady-state well before the timescale of proliferation measurements. The degradation of IGF1R was assumed to be negligible as ELISA analysis demonstrated that down-regulation of IGF1R is small on the timeframe of two hours, which is the time-scale that this model reaches steady-state.

Initial conditions were set to zero for complexes and IGF1 was determined from the treatment conditions. The initial concentration of IGF1R per cell was measured using a total-IGF1R ELISA assay (R&D Systems). To determine the initial concentration of IGFBPs per cell, OVCAR5 were grown in complete medium and then serum-starved for 24 hours to allow for the secretion and accumulation of IGFBPs into the cell culture media. This conditioned media was collected, exogenous IGF1 (0.25 nM) was added and the IGF1-IGFBP interaction was allowed to equilibrate for 2 hours at room temperature. The amount of free IGF1 was determined using the IGF1 ELISA assay, and the steady-state concentration of IGF1-IGFBP complex was determined from the difference between the total IGF1 added and the free IGF1 measured. The amount of free IGFBPs at steady-state was then determined from the steady-state solution to the IGF1-IGFBP interaction:

(7) $$C_{\mathrm{BP}}=\frac{K_{d}\;C_{1:\mathrm{BP}}}{C_{1}}$$

The total level of IGFBPs was determined by summing the amount of IGF1-IGFBP complexes and free IGFBPs at steady-state. The system of equations 1a-e was numerically integrated using an implicit Runge–Kutta method implemented in MATLAB v7.14 (MathWorks, Natick, MA) to calculate the theoretical steady-state concentration of IGF1-IGF1R complexes. Initial conditions and rate coefficient values used in the model are provided in Table 1.

### Model analysis of impact of IGF1 and IGF1R antibodies

To analyze the effects of the addition of an antibody that binds IGF1 or an antibody that binds IGF1R, the model equations were modified as follows. For the inclusion of the IGF1-neutralizing antibody, the IGF1 concentration equation becomes

(8) $$\frac{dC_{1}}{\mathrm{dt}}=k_{-1}C_{1:1R}+k_{-2}C_{1:\mathrm{BP}}-k_{1}C_{1}C_{1R}-k_{2}C_{1}C_{\mathrm{BP}}-k_{3}C_{1}+k_{-4}C_{1:\mathrm{Ab}}-k_{4}C_{1}C_{\mathrm{Ab}}$$

where *k*_*−4*_ and *k*_*4*_ are the rate coefficients for binding and unbinding of IGF1 to the antibody and *C*_*1:Ab*_ and *C*_*Ab*_ are the concentrations of the IGF1:antibody complex and the free antibody. Additionally, the following two new equations were necessary for tracking the concentrations of the antibody and the IGF1:antibody complex.

(9) $$\frac{dC_{\mathrm{Ab}}}{\mathrm{dt}}=k_{-4}C_{1:\mathrm{Ab}}-k_{4}C_{1}C_{\mathrm{Ab}}$$

(10) $$\frac{dC_{1:\mathrm{Ab}}}{\mathrm{dt}}=k_{4}C_{1}C_{\mathrm{Ab}}-k_{-4}C_{1:\mathrm{Ab}}$$

A similar approach was used to include the IGF1R-blocking antibody. The IGF1R concentration evolution equation becomes

(11) $$\frac{dC_{1R}}{\mathrm{dt}}=k_{-1}C_{1:1R}-k_{1}C_{1}C_{1R}+k_{-5}C_{1R:\mathrm{Ab}}-k_{5}C_{1R}C_{\mathrm{Ab}}$$

where *k*_*−5*_ and *k*_*5*_ are the rate coefficients for binding and unbinding of IGF1R to the antibody and *C*_*1R:Ab*_ and *C*_*Ab*_ are the concentrations of the IGF1R:antibody complex and the free antibody. The equations for the evolution of the antibody and the IGF1R:antibody complex are provided below.

(12) $$\frac{dC_{\mathrm{Ab}}}{\mathrm{dt}}=k_{-5}C_{1R:\mathrm{Ab}}-k_{5}C_{1R}C_{\mathrm{Ab}}$$

(13) $$\frac{dC_{1R:\mathrm{Ab}}}{\mathrm{dt}}=k_{5}C_{1R}C_{\mathrm{Ab}}-k_{-5}C_{1R:\mathrm{Ab}}$$

To examine the effects of both antibody binding affinity and treatment dose, we utilized a test matrix that consists of three antibody *K*_*d*_ values (0.1, 1, and 10 nM) and four initial concentrations of the antibody. This test matrix for IGF1R and IGF1 antibodies was applied to several different scenarios. First, the level of IGF1 was varied between low (0.1 nM) and high (2.5 nM) IGF1 conditions while all other test conditions (*i.e.,* initial levels of IGF1R and IGFBPs) were taken from the 67,000 cells/well experimental conditions. To examine the effects of IGFBPs on the results of these calculations, these four scenarios were repeated for IGFBP levels equivalent to 0.1 and 10 times the measured IGFBP level. Finally, to examine the effect of IGF1R levels, these four scenarios were repeated for IGF1R levels equivalent to 10 times the measured IGF1R level. For each of the test scenarios, the output collected from the test matrix was the steady-state concentration of the IGF1-IGF1R complex, which was then used to predict the resulting change in OVCAR5 cell proliferation for each scenario.

### Statistical analysis

All data are presented as the mean ± standard deviation. Statistical significance was evaluated using Tukey’s HSD, with p < 0.05. All statistical calculations were performed using the software package JMP 4.1 (SAS Institute, Cary, NC).

### Consent

Patients were not utilized in this study, therefore the authors have no consent information to report.

## Abbreviations

EGF: Epidermal growth factor

IGF1: Insulin-like growth factor 1

IGF2: Insulin-like growth factor 2

IGFBPs: Insulin-like growth factor binding proteins

IGF1R: Type 1 insulin-like growth factor receptor

IR: Insulin receptor

TGF-α: Transforming growth factor α

TGF-β: Transforming growth factor β

## Competing interests

Both authors declare that they have no competing interests.

## Authors’ contributions

DT participated in the design of the study, performed the experimental analysis, developed and analyzed the model, and drafted the manuscript. PKK conceived of the study, participated in its design and coordination, and drafted the manuscript. Both authors read and approved the final manuscript.

## Additional files

## Supplementary Material

Additional file 1: **Different volumes of cell culture media did not significantly affect proliferation in vehicle-treated OVCAR5.** Baseline proliferation was unaffected by the different volumes of cell culture media used to obtain various dose and concentration combinations. n = 3 per treatment, p > 0.05. Click here for file

Additional file 2: **IGFBP sequestration did not impact initial IGF1 depletion in fresh media.** To isolate the effects of cell-mediated endocytosis and IGFBP sequestration on depletion of IGF1 from cell culture media, OVCAR5 were treated with cycloheximide to block the production of IGFBPs. Cells were pre-treated for 5 hours with 2.5 μg/mL of cycloheximide (Thermo Fisher Scientific, Waltham, MA) or vehicle (water), treated with 0.25 nM IGF1 and cycloheximide or vehicle, and the amount of free IGF1 in the culture media was determined by IGF1 ELISA. The amount of free IGF1 was similar between cycloheximide and control conditions until 4 hours, indicating that IGFBP accumulation in cell culture media was not significant until later times. *indicates significant difference (p < 0.05) from vehicle control. n = 3 per treatment. Click here for file

Additional file 3: **Analysis of relative inhibition of IGF1-neutralizing antibody compared to IGF1R-blocking antibody indicated that IGF1R-blocking antibodies will be more effective.** A range of antibody dissociation constants (*K*_*d*_, 0.1-10 nM) were used to simulate the effect of high to low binding affinity. The effects of the antibody in the presence of three different IGFBP concentrations at *A,* low (0.1 nM) or *B,* high (2.5 nM) IGF1 level were determined using the steady-state model. An intensity greater than 1 indicates that an IGF1-neutralizing antibody would have a stronger inhibitory effect and an intensity less than 1 indicates that the IGF1R-blocking antibody would be more effective. Model results indicated that an antibody that blocks IGF1R would more strongly inhibit IGF1-induced cell proliferation. Click here for file

Additional file 4: **Model-predicted reduction in cell proliferation in response to antibody treatment indicated that IGF1R-blocking antibodies will be more effective than IGF1-neutralizing antibodies, even with elevated IGF1R levels.** A range of antibody dissociation constants (*K*_*d*_, 0.1-10 nM) were used to simulate the effect of high to low binding affinity for conditions where the initial concentration of IGF1R was increased by a factor of 10. The effects of the antibody in the presence of three different IGFBP concentrations at *A,* low (0.1 nM) or *B,* high (2.5 nM) IGF1 level were determined using the steady-state model. Model results indicated that an antibody that blocks IGF1R would more strongly decrease the steady-state concentration of IGF1-IGF1R complexes and consequently, inhibit IGF1-induced cell proliferation, than an antibody that binds and neutralizes IGF1. Model results also indicated that increasing the initial receptor concentration by a factor of 10 (relative to Figure 4) had little impact because the concentration of IGF1-IGF1R complexes was most strongly limited by available IGF1R. Click here for file

Additional file 5: **Protein complex immunoprecipitation (IP) determined that the IGF1R/IR heterodimer population was a small fraction of total IGF1R receptors in OVCAR5.** IP analysis of IGF1R/IR was evaluated using IGF1R and IR antibody as the bait. The presence of IGF1R/IR in OVCAR5 was determined to be less than 10% of the total IGF1R population. OVCAR5 were grown to 80% confluency and lysed with non-reducing lysis buffer composed of 1% NP-40 Alternative (EMD Biosciences, La Jolla, CA), 20 mM Tris (pH 8.0), 137 mM NaCl, 10% glycerol, 2 mM EDTA (Boston BioProducts, Ashland, MA), 1 mM sodium orthovanadate, 10 μg/mL aprotinin, 10 μg/mL leupeptin, 1 μg/mL pepstatin, and 1 mM PMSF. Total protein was measured by BCA assay. Equal amounts of protein (200 μg) was added to IGF1R (1:100) or IR (1:50) antibody and incubated overnight at 4°C with gentle shaking. Protein A Agarose (Pierce, Rockford, IL) was added to the mixture and incubated at 4°C with gentle rocking for 3 hours. The mixture was then centrifuged at 4°C at 18.8 g for 30 seconds and the pellet was then gently washed using non-reducing lysis buffer. The pellet was then separated by SDS–PAGE and blotted onto nitrocellulose membranes. Membranes were incubated overnight at 4°C in primary antibody and for 1 hour at room temperature in secondary antibody. Membranes were washed and fluorescence signals were detected and quantified using the Odyssey Infrared Imaging System (LI-COR Biotechnology, Lincoln, NE). Anti-IGF1R (1:1000; #9750), anti-IR (1:1000; #3020), and GAPDH (1:10,000; #2118) were purchased from Cell Signaling Technology (Devers, MA). Secondary antibodies were purchased from LI-COR Biotechnology and used at 1:15,000 dilution. The starting lysate control where the lysate was not subjected to IP was loaded at 25 μg per lane, representing 1/8 of the total protein concentration used for IP. n = 3 per treatment. Click here for file

Additional file 6: **OVCAR5 proliferation in response to IGF1 was dependent on IGF1R kinase activity.** Cells were pre-treated with an IGF1R tyrosine kinase inhibitor (NVP-AEW541, 1 μM) or an IR tyrosine kinase inhibitor (HNMPA-(AM_3_), 5 μM) for 30 minutes before stimulation with a saturating dose of IGF1 (13 nM) for 24 hours. The results demonstrated that IGF1R kinase activity was essential for OVCAR5 proliferation in response to IGF1, while IR kinase activity was not. *indicates significantly different (p < 0.05) from IGF1-treated, n = 3 per treatment. Click here for file
