# Supplementary figures and images for: Analysis of the quantitative balance between insulin-like growth factor (IGF)-1 ligand, receptor, and binding protein levels to predict cell sensitivity and therapeutic efficacy
Source: BMC Syst Biol. 2014 Aug 13;8:98. doi: 10.1186/s12918-014-0098-y (PMC4236724; doi:10.1186/s12918-014-0098-y)

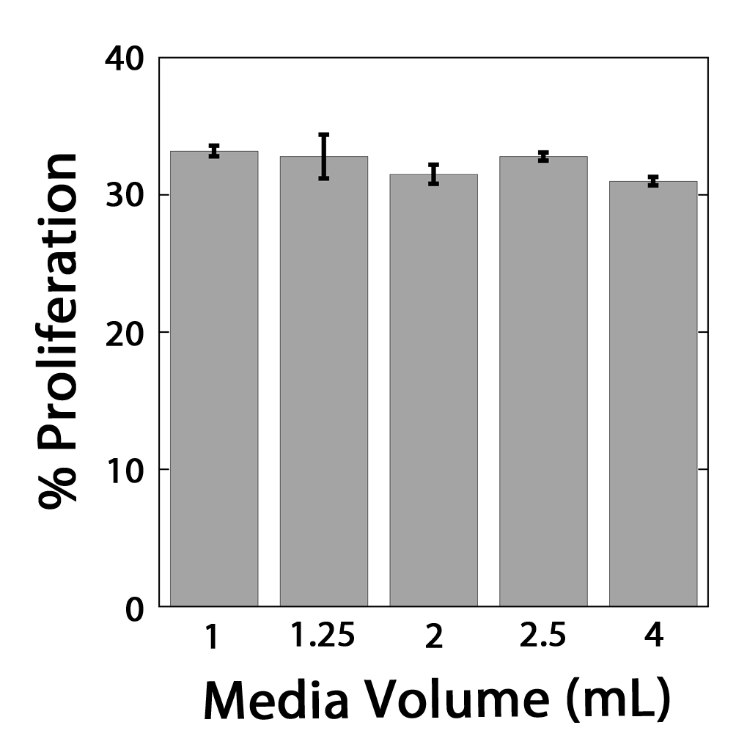

Supplement: Additional file 1: — Different volumes of cell culture media did not significantly affect proliferation in vehicle-treated OVCAR5. Baseline proliferation was unaffected by the different volumes of cell culture media used to obtain various dose and concentration combinations. n = 3 per treatment, p > 0.05. [file s12918-014-0098-y-S1.docx]

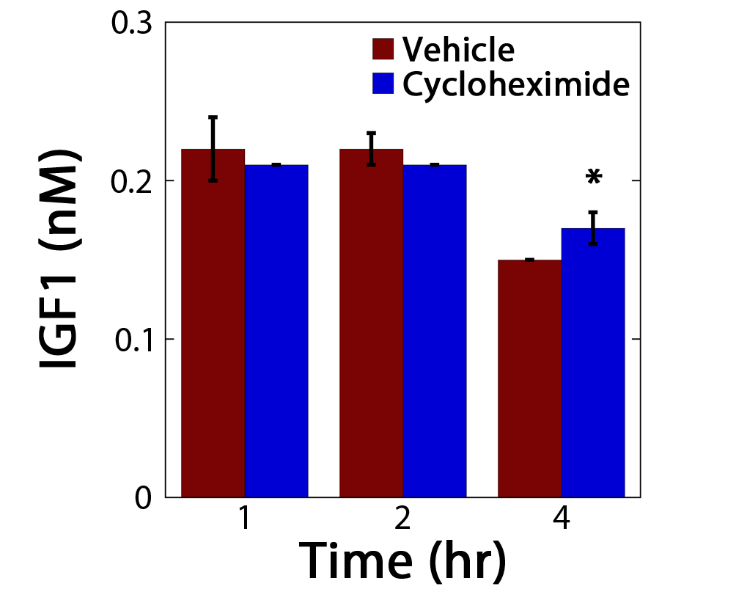

Supplement: Additional file 2: — IGFBP sequestration did not impact initial IGF1 depletion in fresh media. To isolate the effects of cell-mediated endocytosis and IGFBP sequestration on depletion of IGF1 from cell culture media, OVCAR5 were treated with cycloheximide to block the production of IGFBPs. Cells were pre-treated for 5 hours with 2.5 μg/mL of cycloheximide (Thermo Fisher Scientific, Waltham, MA) or vehicle (water), treated with 0.25 nM IGF1 and cycloheximide or vehicle, and the amount of free IGF1 in the culture media was determined by IGF1 ELISA. The amount of free IGF1 was similar between cycloheximide and control conditions until 4 hours, indicating that IGFBP accumulation in cell culture media was not significant until later times. *indicates significant difference (p < 0.05) from vehicle control. n = 3 per treatment. [file s12918-014-0098-y-S2.docx]

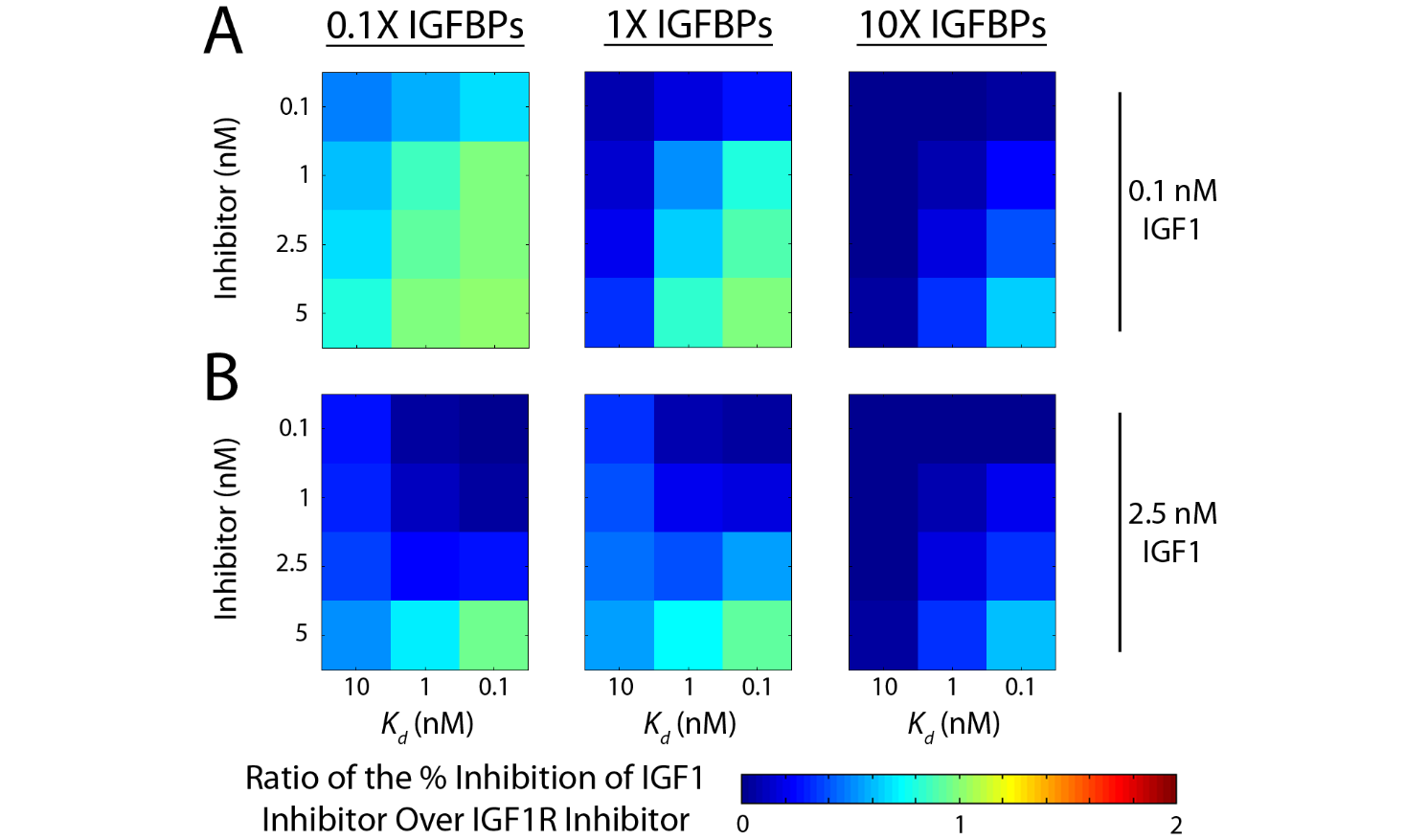

Supplement: Additional file 3: — Analysis of relative inhibition of IGF1-neutralizing antibody compared to IGF1R-blocking antibody indicated that IGF1R-blocking antibodies will be more effective. A range of antibody dissociation constants (Kd, 0.1-10 nM) were used to simulate the effect of high to low binding affinity. The effects of the antibody in the presence of three different IGFBP concentrations at A, low (0.1 nM) or B, high (2.5 nM) IGF1 level were determined using the steady-state model. An intensity greater than 1 indicates that an IGF1-neutralizing antibody would have a stronger inhibitory effect and an intensity less than 1 indicates that the IGF1R-blocking antibody would be more effective. Model results indicated that an antibody that blocks IGF1R would more strongly inhibit IGF1-induced cell proliferation. [file s12918-014-0098-y-S3.docx]

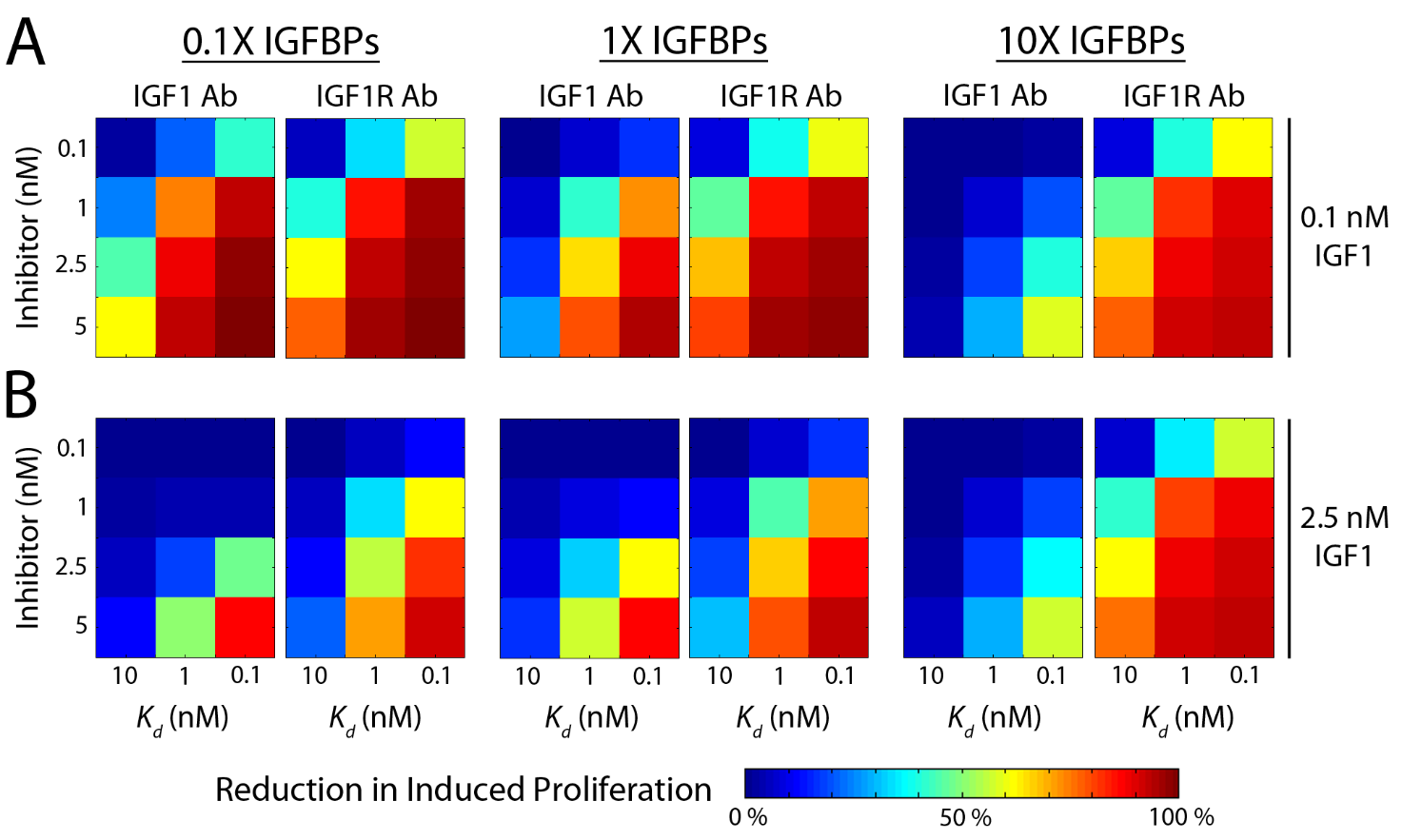

Supplement: Additional file 4: — Model-predicted reduction in cell proliferation in response to antibody treatment indicated that IGF1R-blocking antibodies will be more effective than IGF1-neutralizing antibodies, even with elevated IGF1R levels. A range of antibody dissociation constants (Kd, 0.1-10 nM) were used to simulate the effect of high to low binding affinity for conditions where the initial concentration of IGF1R was increased by a factor of 10. The effects of the antibody in the presence of three different IGFBP concentrations at A, low (0.1 nM) or B, high (2.5 nM) IGF1 level were determined using the steady-state model. Model results indicated that an antibody that blocks IGF1R would more strongly decrease the steady-state concentration of IGF1-IGF1R complexes and consequently, inhibit IGF1-induced cell proliferation, than an antibody that binds and neutralizes IGF1. Model results also indicated that increasing the initial receptor concentration by a factor of 10 (relative to Figure 4) had little impact because the concentration of IGF1-IGF1R complexes was most strongly limited by available IGF1R. [file s12918-014-0098-y-S4.docx]

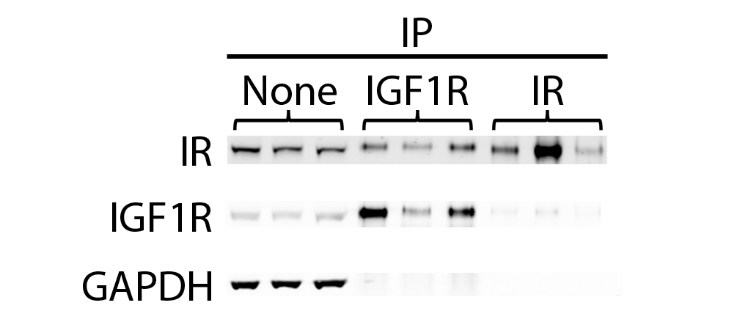

Supplement: Additional file 5: — Protein complex immunoprecipitation (IP) determined that the IGF1R/IR heterodimer population was a small fraction of total IGF1R receptors in OVCAR5. IP analysis of IGF1R/IR was evaluated using IGF1R and IR antibody as the bait. The presence of IGF1R/IR in OVCAR5 was determined to be less than 10% of the total IGF1R population. OVCAR5 were grown to 80% confluency and lysed with non-reducing lysis buffer composed of 1% NP-40 Alternative (EMD Biosciences, La Jolla, CA), 20 mM Tris (pH 8.0), 137 mM NaCl, 10% glycerol, 2 mM EDTA (Boston BioProducts, Ashland, MA), 1 mM sodium orthovanadate, 10 μg/mL aprotinin, 10 μg/mL leupeptin, 1 μg/mL pepstatin, and 1 mM PMSF. Total protein was measured by BCA assay. Equal amounts of protein (200 μg) was added to IGF1R (1:100) or IR (1:50) antibody and incubated overnight at 4°C with gentle shaking. Protein A Agarose (Pierce, Rockford, IL) was added to the mixture and incubated at 4°C with gentle rocking for 3 hours. The mixture was then centrifuged at 4°C at 18.8 g for 30 seconds and the pellet was then gently washed using non-reducing lysis buffer. The pellet was then separated by SDS–PAGE and blotted onto nitrocellulose membranes. Membranes were incubated overnight at 4°C in primary antibody and for 1 hour at room temperature in secondary antibody. Membranes were washed and fluorescence signals were detected and quantified using the Odyssey Infrared Imaging System (LI-COR Biotechnology, Lincoln, NE). Anti-IGF1R (1:1000; #9750), anti-IR (1:1000; #3020), and GAPDH (1:10,000; #2118) were purchased from Cell Signaling Technology (Devers, MA). Secondary antibodies were purchased from LI-COR Biotechnology and used at 1:15,000 dilution. The starting lysate control where the lysate was not subjected to IP was loaded at 25 μg per lane, representing 1/8 of the total protein concentration used for IP. n = 3 per treatment. [file s12918-014-0098-y-S5.docx]

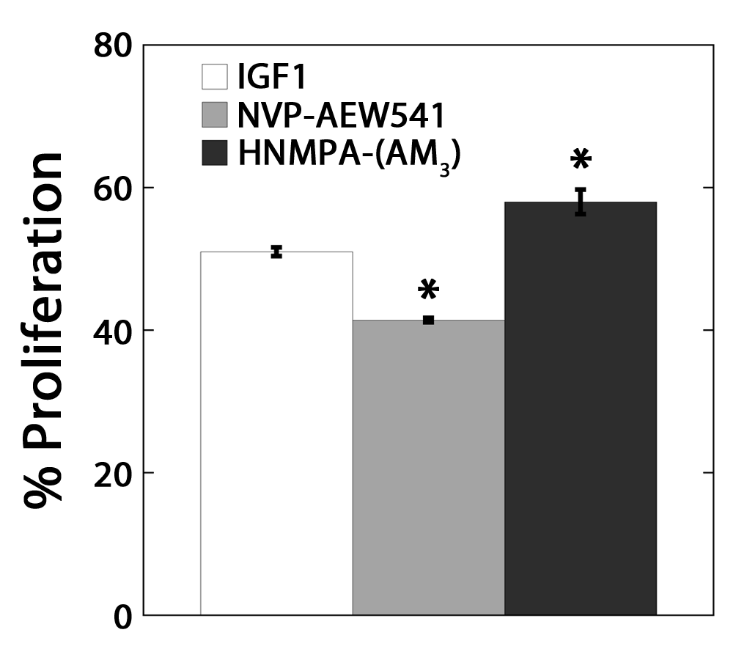

Supplement: Additional file 6: — OVCAR5 proliferation in response to IGF1 was dependent on IGF1R kinase activity. Cells were pre-treated with an IGF1R tyrosine kinase inhibitor (NVP-AEW541, 1 μM) or an IR tyrosine kinase inhibitor (HNMPA-(AM3), 5 μM) for 30 minutes before stimulation with a saturating dose of IGF1 (13 nM) for 24 hours. The results demonstrated that IGF1R kinase activity was essential for OVCAR5 proliferation in response to IGF1, while IR kinase activity was not. *indicates significantly different (p < 0.05) from IGF1-treated, n = 3 per treatment. [file s12918-014-0098-y-S6.docx]
